# Ecological niches and blood sources of sand fly in an endemic focus of visceral leishmaniasis in Jiuzhaigou, Sichuan, China

**DOI:** 10.1186/s40249-016-0126-9

**Published:** 2016-04-13

**Authors:** Huiying Chen, Kaili Li, Hua Shi, Yong Zhang, Yu Ha, Yan Wang, Jinjin Jiang, Yubin Wang, Zhenzhou Yang, Jiannong Xu, Yajun Ma

**Affiliations:** Department of Tropical Infectious Diseases, Faculty of Tropical Medicine and Public Health, Second Military Medical University, Shanghai, 200433 China; Biology Department, Molecular Biology Program, New Mexico State University, Las Cruces, NM 88003 USA; Center for Disease Control and Prevention of PLA, Beijing, 100071 China; Jiuzhaigou Center of Disease Control and Prevention, Jiuzhaigou, Sichuan 623400 China; Center of Disease Control and Prevention, General Equipment Department of PLA, Beijing, 100101 China

**Keywords:** Sand fly, *Phlebotomus chinensis*, Ecological niche, Blood sources, China

## Abstract

**Background:**

Sand fly *Phlebotomus chinensis* is a principle vector for the visceral leishmaniasis (VL) in China with a wide geographic distribution. Jiuzhaigou, Sichuan is a mountain type endemic area of VL in China. Long term effective control efforts in the region have successfully reduced VL transmission. To assess the current status of the sand flies and their ecological aspects in the region, a survey was conducted in the summer of 2014 and 2015.

**Methods:**

Sand fly specimens were collected by light traps in a village and blood sources were identified by PCR and sequencing of the mitochondrial cytochrome *b* gene.

**Results:**

In a rock cave, 65.2 %–79.8 % of collected sand flies were male. On a rabbit farm, 92.9 %–98.8 % of specimens were female. In pig pens, 61.1 % of specimens were female. Some females had visible blood residues. The feeding rate was 49.4 % from the pig pens, 12.3 % from the cave, and only 1.7 % from the rabbit farm. Pig, rabbit, chicken, dog, and human blood were detected in the fed specimens. Swine blood, present in all tested samples, was a preferred blood source, while chicken and dog blood were present in a third of the samples.

**Conclusions:**

In Jiuzhaigou County, Sichuan Province of China, the considerable sandfly density and the peridomestic feeding behavior all increases the risk of VL transmission, and insecticide spraying in animal sheds could be exploited to reduce sand fly populations in human surroundings.

**Electronic supplementary material:**

The online version of this article (doi:10.1186/s40249-016-0126-9) contains supplementary material, which is available to authorized users.

## Multilingual abstracts

Please see additional file 1 for translation of the abstract into the six official working languages of the United Nations.

## Background

Visceral leishmaniasis (VL) is a disease caused by trypanosomatid protozoa in the genus *Leishmania* and is transmitted by vector species of phlebotomine sand flies. At present, VL is largely endemic in western China; focal and sporadic cases occur in Xinjiang, Inner Mongolia, Gansu, Sichuan, Shaanxi, and Shanxi [1, 2]. Jiuzhaigou is one of the VL endemic foci in Sichuan Province, and sand flies in the region have been investigated since the 1980s [3–6]. Five species of sand flies exist in the area: *Phlebotomus chinensis*, *Ph. sichuanensis, Sergentomyia quamirostris*, *S. suni*, and *S. koloshanensis. Phlebotomus chinensis* is the most abundant species, accounting for 96 % of the sand fly population [5, 6]. Annually, sand flies emerge in May, peak between late July to early August, and then decline in September and disappear by late October. They are largely exophilic and are commonly found in rock and dirt caves [5, 6]. Epidemiologically, VL in Sichuan is zoonotic, maintained in cycles between animals and sand flies [4]. *Phlebotomus chinensis* is the principle vector and domestic dogs are the primary reservoir host. Natural infection of *Leishmania* was detected in wild caught sand fly females with 1.98 % prevalence [7]. The prevalence of *Leishmania* infection in dogs is high in the region. In two surveys conducted in 2010, the infection rate of *Leishmania* in dogs at Jiuzhaigou was 59.4 % [8] and 24.1 % [9].

Integrated implementations of control efforts in the past decades have greatly reduced the prevalence and incidence of VL in China [2]. In Jiuzhaigou, VL has declined from 60–70 cases a year in the 1970s to less than 10 cases a year in the year 2010–2014. The successful reduction of VL in the region was largely attributed to the control and treatment of infected dogs. Both veterinary care and insecticide-impregnated collars effectively intervened the VL transmission. However, the risk of VL remains due to the existence of wild animal reservoirs and sand flies. Jiuzhaigou is a famous scenic attraction for tourists with approximately 4.5 million visitors in 2014 according to a press release by Jiuzhaigou Tourism Bureau. The non-immune tourists are vulnerable, and risks of contracting VL are persistently present. Surveillance and control of sand flies have become an urgent necessity in the local VL control program. Understanding the current status of the bionomics of sand flies will facilitate development of effective control measures. In this paper, we report the habitat types and blood sources of sand flies in the region.

## Methods

### Ethical statement

This study was carried out in strict accordance with the NSFC, NIH and NMSU ethical guidelines for biomedical research involving living animals and human subjects.

### Sand fly collection and species identification

**Fig. 1:**
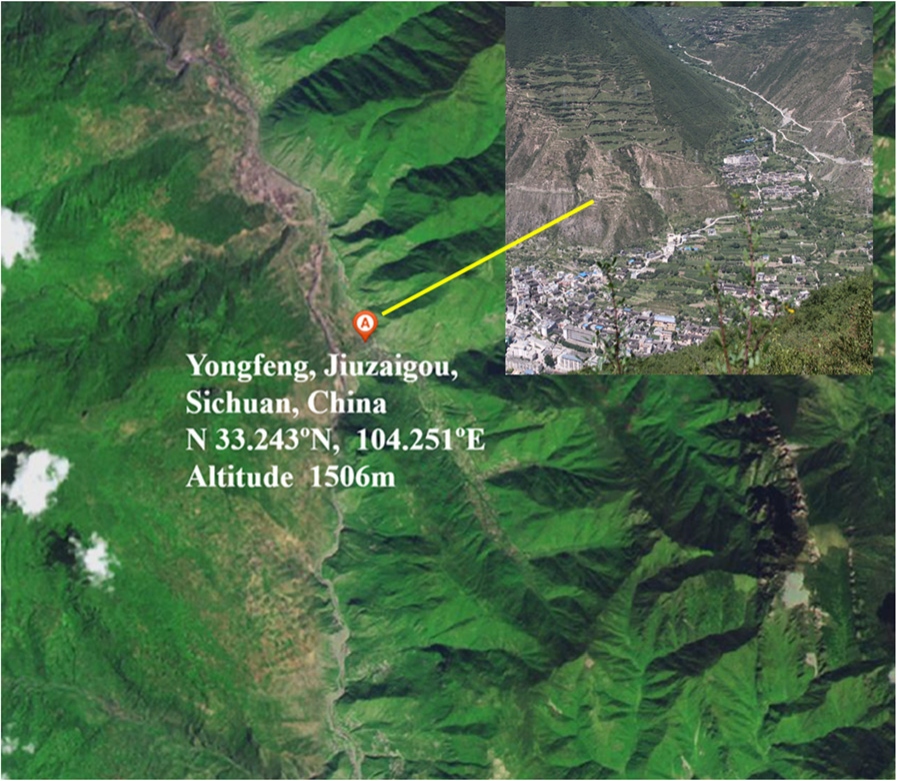
The landscape of the collection sites, Jiuzhaigou County, Sichuane Province, China

The sand fly specimens were collected in Shangzhai Village, Yongfeng, Jiuzhaigou County, Sichuan Province, China in July of 2014 and 2015 (Fig. 1). The village is located at an altitude 1 200–1 600 m along a valley. Houses are built into the hillside. CDC mini light traps (BioQuip, USA) and light traps (Shengzhen, China) were used to catch sand flies. With the owners’ consent, the light traps were set up in a cave, a rabbit house, and three pig pens between 6:30 pm-8:30 am. Specimens of *Phlebotomus chinensis* were recognized by morphological keys [10]. Specimens with visible blood residues were used for blood source identification. The specimens were preserved in RNAfixer (Aidlab Biotechnologies Co., Ltd, China) and brought back to the lab for DNA isolation. The DNA was isolated using DNAzol (Life Technologies, USA), following the manufacturer’s instruction. The identity of *Ph. chinensis* was verified by sequencing rDNA PCR products from 20 randomly selected single specimens using a previously developed assay [11].

### Blood source identification

PCR based detection of mitochondrial *cytochrome b* gene fragment has been used for identifying blood source in sand flies [12–14]. Therefore, PCR and sequencing of mitochondrial *cytochrome b* genes from human, cow, pig, dog, chicken and rabbit were used for identifying blood sources in the sand fly specimens. The primers used are listed in Table 1. To get sufficient DNA for several PCR reactions, 9 or 10 fed individuals from each collection site were pooled together as one sample. DNA was isolated from each sample using DNAzol, and DNA pellet was dissolved in 50 μl H_2_O. The PCR reaction was run in a 25 μl mix including 1.5 μl DNA template, 0.2 μM primers, and other PCR reagents (Aidlab Biotechnologies Co., Ltd, China). The cycling parameters were set as denaturing at 94 °C for 15 s, annealing temperature ramping between 51–59 °C for 30 s, extension at 72 °C for 1 min and cycled for 35 times. For each sample, six PCR reactions were performed using species specific primer sets. The PCR products were purified and sequenced at Boshang Biotech (Shanghai, China) Co., Ltd.

**Table 1:** PCR primers for blood source identification

Species	Primers	Sequence (5'to3')	Amplicon length (bp)
Universal reverse	UR	GGT TGT CCT CCA ATT CAT GTT A	
Human (*Homo sapien*)	HF + UR	GGC TTA CTT CTC TTC ATT CTC TCC T	334
Pig (*Sus domesticus*)	PF + UR	CCT CGC AGC CGT ACA TCT C	453
Cow (*Bos taurus*)	CF + UR	CAT CGG CAC AAA TTT AGT CG	561
Dog (*Canis lupus*)	DF + UR	GGA ATT GTA CTA TTA TTC GCA ACC AT	680
Chicken (*Gallus gallus*)	ChF	CAT ACT CCC TCA CTC CCC CA	802
	ChR	CCC CTC AGG CTC ACT CTA CT	
Rabbit (*Oryctolagus cuniculus*)	RF	CGA TAC CTC CAC GCT AAC GG	388
	RR	TTG GGT TGT TGG AGC CAG TT	

Note: *F* Forward, *R* Reverse. Universal reverse primer was used in couple with respective forward primers

### Statistical analysis

The sex composition was compared between different collections by Chi-Square test, which was conducted by SigmaStat 3.5 (Systat Software Inc.). The feeding rates among different collections were compared by a Chi-Square (*r* × *c*) contingency table, which was implemented at http://www.physics.csbsju.edu/stats/contingency_NROW_NCOLUMN_form.html.

## Results

### Sand fly collections from different ecological niches

**Fig. 2:**
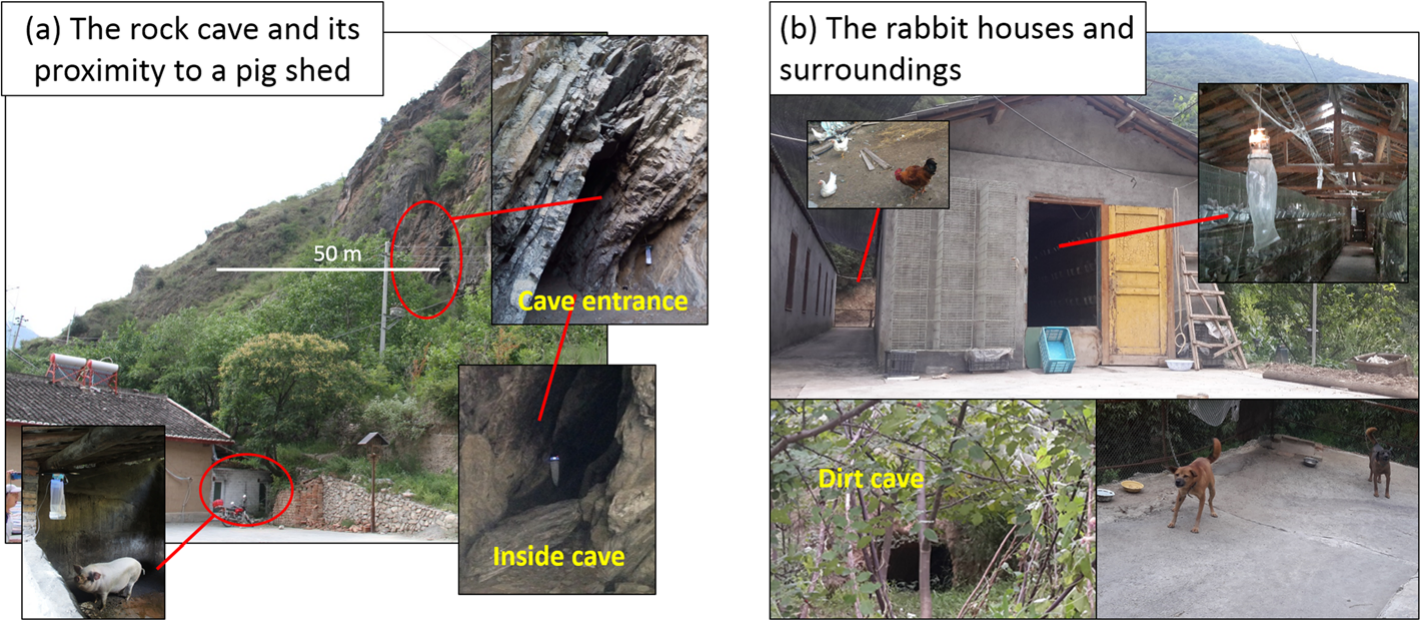
Ecological niches of sand flies in the study site. (**a**) The rock cave and its proximity to a house with a pig pen. (**b**) The rabbit farm, dogs and chickens, and a nearby dirt cave

Sand flies of *Ph. chinensis* were collected in Jiuzhaigou County, Sichuan, China in July, 2014 and July, 2015. In the village, many households own dogs. Pig pens were usually built adjacent to human houses or in close proximity. Chickens are raised in the yard. In the summer, horses and cattle were moved into the mountains to graze, so these domestic animals were not present in the village when the sand flies were collected. Light traps were installed in a rock cave near the village, a rabbit farm and three pig pens in the village. The rock cave was a well-known sand fly habitat with data back to the 1980s [4], 1990s [6] and 2000s [5]. The cave was located at altitude 1 500 m, with dimension of (2–2.5) m × 12 m. The straight-line distance between the cave and the nearest house was about 50 m (Fig. 2a). The small rabbit farm had two sheds in which approximately 1 200 rabbits, *Oryctolagus cuniculus*, were housed. The owner lived in a room that was adjacent to one of the sheds. Two dogs were leashed at the entrance to the rabbit farm. Chickens were raised in the back yard. There was a small dirt cave that was located about 10 m away from the rabbit farm (Fig. 2b).

In the 2014 collection, the light traps were setup only in the rock cave and the rabbit sheds. A total of 3 255 specimens were caught in six nights, 2 412 specimens were collected in the cave and 843 were in the rabbit sheds. In the cave, 1 923 (79.8 %) specimens were males while in the rabbit sheds, 833 (98.8 %) specimens were females (Table 2). The sex composition was significantly different between the rabbit sheds and cave (*χ*^2^ = 1594.4, *P* < 0.01). In 2015, 4 492 specimens were caught in five nights. A similar sex composition was observed, more females (1 726/1 858, 92.9 %) were caught in the rabbit sheds, and more males (1 571/2 411, 65.2 %) were caught in the cave, the difference was significant (*χ*^2^ = 1472.5, *P* < 0.01). In addition, light traps were used in three pig pens for three nights, which caught 223 specimens. Females accounted for 61.1 % of the collection. The specimens were defined as fed if blood was visible. The feeding rate was high in the collection from the pig pens (49.4 %), followed by the cave collection (12.3 %). The feeding rate in the rabbit sheds (1.7%) was significantly lower than that in the cave and pig pens (R × C contingency table, *χ*^2^ = 529, *P* < 0.01) (Table 3).

**Table 2:** Sand fly collections in the study

Time	Sites	No. of males (%)	No. of females (%)	Total
July, 2014	Rock cave	1923 (79.8)	489 (20.2)	2 412
	Rabbit sheds	10 (1.2)	833 (98.8)	843
July, 2015	Rock cave	1571 (65.2)	840 (34.8)	2 411
	Rabbit sheds	132 (7.1)	1726 (92.9)	1 858
	Pig pens	45 (38.9)	178 (61.1)	223

**Table 3:** Feeding rates in the 2015 collection

	No. of fed females (%)	Total No. of females
Rock cave	103 (12.3)	840
Rabbit sheds	29 (1.7)	1 726
Pig pens	88 (49.4)	178

### Blood sources

In the 2015 collections, blood origins were determined molecularly. There were 10 samples from 100 fed specimens of the cave collection, 3 samples from 29 fed specimens from the rabbit sheds and 9 samples from 88 fed specimens from the pig pens. For each sample, six distinct PCR reactions were performed to amplify *cyt b* genes from human, pig, chicken, rabbit, dog and cow, respectively. The size of PCR products was animal specific (Table 1), and PCR products from all positive reactions were sequenced to confirm animal identity (data not shown). As shown in Table 4, swine blood was detected in all 22 samples from all of the three collection sites. Chicken blood was found in two samples from the cave, two samples from the rabbit sheds, and four samples from the pig pens. As expected, rabbit blood was detected in all of three samples from the rabbit sheds. Intriguingly, two samples from the cave were positive for the rabbit blood. Human blood was detected only in one sample from the pig pens, and dog blood was positive in six samples from the cave and one sample from the pig pens. Cow blood was not detected in any of these samples as expected.

**Table 4:** Blood sources detected in the fed sand flies

Location	Blood origin					
	(No. of positive samples/total No. of samples)					
	Pig	Rabbit	Chicken	Dog	Human	Cow
Rock cave	10/10	2/10	2/10	6/10	0/10	0/10
Rabbit sheds	3/3	3/3	2/3	0/3	0/3	0/3
Pig pens	9/9	0/9	4/9	1/9	1/9	0/9

## Discussion

Sand flies can adapt to various ecological niches and have quite a broad range of hosts as blood sources [12, 13, 15, 16]. The development of an effective measure of sand fly control would largely rely on the understanding of habitats and host preference in a region. In the study, sand flies were sampled from one large rock cave, two rabbit sheds, and three pig pens; which represented three types of habitats in the region. As shown in Table 2, more sand flies were caught in the cave than in the village (rabbit sheds and pig pens). Evidently, the cave was a good breeding habitat close to the village. Males were predominant in the cave collections, suggesting that males stay primarily at that habitat. Females need to hunt for blood in a larger radius. Consistent with this, 12.3 % of females caught in the cave had visible blood residue. Pig, rabbit, chicken, and dog blood were detected in the cave collection. Apparently females fly into the village to obtain blood and return to the cave to lay eggs. It was particularly interesting that rabbit blood was positive in the flies from the cave. There was only one rabbit farm located approximately 500 m away from the cave. Most likely, the sand flies that took rabbit blood would fly to the cave for oviposition. Alternatively, sand flies could have taken blood from wild hares that were near the cave. Overall, the evidences strongly suggest that the rock cave is an optimal sand fly breeding site near the village.

The rabbit sheds were attractive for females most likely due to the high amount of CO_2_ produced by the large number of rabbits, as CO_2_ has been shown to be an effective attractant for sand flies [17–19]. Interestingly, the feeding rate in the collection was much lower than from the rock cave and pig pens. We do not have an explanation for this phenomenon. The rabbit and hare have been shown to be a blood source for Phlembotomine sand flies [20–25]. In a focus of leishmaniasis in the southwestern Madrid region, Spain [22], rabbits may play a role in the transmission of *Leishmania infantum* to *Ph. perniciosus* [23]. Potentially, rabbit farming may pose a risk in leishmaniasis endemic areas.

In the collection from pig pens, greater than half specimens were females, and half of the females were engorged. As expected, swine blood was found in all 9 samples from pig pens. In addition, swine blood was found in all 13 samples from the cave and rabbit sheds. It appears that sand flies preferred to take blood from pigs. Pigs have been reported to be a blood source for sand flies [26]. In addition, the soil in the pig pens enriched with organic compounds released from swine excretions may provide supports for larval development [27, 28]. A *Leishmania* infected pig has been documented. The *Leishmania* amastigotes were detected in the cutaneous lesion [29], which left a possibility that pigs might be able to sustain cutaneous infections. In a study conducted in a region of Brazil where American visceral leishmaniasis was endemic, the prevalence of antibodies against *L. infantum* were about 40 % in the pigs tested. However, *when sows were experimentally inoculated with infective L. infantum promastigotes, anti- L. infantum* antibody was induced, but no full infection was established. [30]. The data suggest that pigs are able to develop effective immunity to eliminate *L. infantum* infection. The immunity of pigs against *L. infantum* infection greatly reduces the possibility of serving as a reservoir host for *L. infantum.* Recently, multiple lines of evidence suggest that there are heterogeneous *Leishmania* strains in China. These strains are distinct from but phylogenetically related to *L. donovani/L. infantum* complex [7, 31–34]. Therefore, further study is needed to investigate whether or not pigs can serve as a reservoir host of the *Leishmania* strains in China.

## Conclusions

The presence of various ecological niches and the availability of ample blood sources from domestic animals contributed to the maintenance of a large population of sand flies. Habitats such as rock caves in the vicinity and peridomestic pigsties should be included in sand fly control. Sand flies in the region were susceptible to insecticides. In 1994, the rock cave was treated with alpha-cypermethrin. The treatment eliminated the sand flies in the cave instantly, and no sand flies were found for four consecutive years [6]. Therefore, spraying residual insecticides inside rock caves and pig pens may be an affordable and sustainable method for reducing the sand fly populations in and around human living quarters.

## Additional file

Additional file 1: Multilingual abstracts in the six official working languages of the United Nations. (PDF 289 kb)
