# Supplementary material for: Ecological niches and blood sources of sand fly in an endemic focus of visceral leishmaniasis in Jiuzhaigou, Sichuan, China
Source: Infect Dis Poverty. 2016 Apr 13;5:33. doi: 10.1186/s40249-016-0126-9 (PMC4831150; doi:10.1186/s40249-016-0126-9)
Supplement: Additional file 1: — Multilingual abstracts in the six official working languages of the United Nations. (PDF 289 kb) [file 40249_2016_126_MOESM1_ESM.pdf]

Translation of the abstract into the six official working languages of the United Nations

كوات إيكولوجية ومصادر الدم من ذُبابة الرمل في بؤرة مستوطن داء الليشمانيات الحشوي في مدينة جيوتشايجو، سيشوان، الصين

ينغ تشن، كايلي لي، هوا شي، يونغ تشانغ، يو ها، يان وانغ، جينجين، جيانغ، شيانغ يو لي، يوبين وانغ، تشنغتشو يانغ، جيانونج شو، ياجون ما

ملخص

معلومات أساسية ذبابة الرمال "الفصيلة الصينية" هي ناقل رئيسي داء الليشمانيات الحشوي (VL) في الصين على نطاق جغرافي واسع. تعد مقاطعة جيوتشايقو الواقعة في مدينة سيشوان منطقة ذات طابع جبلي وتعد مستوطنًا لداء الليشمانيات الحشوي (VL) في الصين. لقد نجحت جهود المكافحة الفعالة طويلة الأجل في المنطقة لخفض انتقال داء الليشمانيات الحشوي. لقد أجريت دراسة استقصائية في صيف 2014 و 2015 لتقييم الوضع الحالي لذباب الرمل والجوانب البيئية الخاصة به.

الطرق تم جمع عينات من ذبابة الرمل بواسطة الفخاخ الخفيفة في قرية ومصادر الدم التى تم تحديدها بواسطة تفاعل البوليميريز المتسلسل "PCR" وتسلسل جينات الستوكروم "ب" الميتوكونديرى."Mitochondrial Cytochrome b Genes"

النتائج وجد في كهف صخري قريب أن نسبة 65.2% - 79.8% من ذباب الرمل الذي تم جمعه من الذكور. كما وجد أن نسبة 92.9% - 98.8% من العينات التى أخذت من مزرعة الأرانب كانت من الإناث. كما أظهرت النتائج أن نسبة 61.1% من العينات التي تم أخذها من مزرعة للخنازير كانت من الإناث. وكان لبعض الإناث بقايا دم مرئية. كان معدل التغذية 49.4% في الإناث من حظائر الخنازير، و 12.3% من الكهف، و 1.7% فقط من مزرعة الأرانب. تم اكتشاف دم خنزير، ودجاج، وكلب وإنسان في عينات التغذية. وكان دم الخنزير موجودًا في جميع العينات المُختبرة، ويعد مصدر دم مُفضل لذباب الرمال، بينما كان كلا من دم الدجاج والكلب موجودان في ثلث العينات.

الاستنتاجات في مقاطعة جيوتشايجو، وإقليم سيشوان الصيني، أدت زيادة كثافة ذباب الرمل بشكل كبير وسلوك التغذية إلى ويادة مخاطر انتقال داء الليشمانيات الحشويVL ، ووجد أن رش المبيدات الحشرية في حظائر الحيوانات يمكن استخدامة لتقلق أعداد ذبابة الرمل في المحيطات المأهولة بالسكان.

Translated from English version into Arabic by Alain Alameddine, through

TRANSLATORS
WITHOUT BORDERS

中国四川九寨沟白蛉的生态习性调查和血源动物鉴定研究

陈辉莹，李凯利，石华，张勇，哈煜，王琰，李翔宇，蒋金津，王育兵，杨振洲，徐建农，马雅军

摘要

引言：中华白蛉在中国分布广泛，是内脏利什曼病的主要传播媒介。四川省九寨沟县是中国内脏利什曼病的山区型流行区，长期有效地防控措施实施使疫情得到控制，为评估当地传播媒介白蛉的现状，作者于 2014 年和 2015 年对其生态习性进行了调查，并鉴定白蛉的血源动物。

方法：在四川省九寨沟县上寨村周围的各种环境灯诱法采集标本，基于线粒体 DNA 细胞色素 b 基因的序列，PCR 分子鉴定白蛉的血源动物种类。

结果：在村庄附近的石洞，采集到的白蛉中雄性的占 65.2%-79.8%，而在兔圈中，雌性白蛉所占比例高达 92.9%-98.8%，在猪圈中采集的雌蛉占总数的 61.1%；在采集的雌蛉中，吸血的个体所占比例，在猪圈中比例最高为 49.4%，石洞为 12.3%，兔圈仅 1.7%。对吸血的白蛉进行血源动物鉴定，结果显示有猪、兔、鸡、狗，还有人；其中猪在每组样本中均可检测，而鸡和狗仅在 3 组样本中可检测。

结论：四川省九寨沟县的白蛉密度较大，且吸血对象广泛，增加了当地的内脏利什曼病传播的风险，可通

过在牲畜圈中喷洒杀虫剂减少人居环境中的白蛉种群。

Translated from English version into Chinese by Ma Yajun.

Niches écologiques et sources de sang du phlébotome dans un foyer de leishmaniose viscérale endémique à Jiuzhaigou (Sichuan, Chine)

Huiying Chen, Kaili Li, Hua Shi, Yong Zhang, Yu Ha, Yan Wang, Jinjin Jiang, Xiangyu Li, Yubin Wang, Zhenzhou Yang, Jiannong Xu, Yajun Ma

Résumé

Contexte: Principal vecteur de la leishmaniose viscérale en Chine, le phlébotome *Phlebotomus chinensis* est présent dans une vaste aire de distribution géographique. Jiuzhaigou, dans le Sichuan, est une zone d'endémie de leishmaniose viscérale de type montagneux. Des mesures prolongées et efficaces de lutte contre le vecteur ont permis d'y réduire la transmission de la leishmaniose viscérale. Une étude a été menée pendant les étés 2014 et 2015 pour évaluer le statut actuel des phlébotomes et leurs paramètres écologiques dans la région.

Méthodes: Des spécimens de phlébotomes ont été collectés à l'aide de pièges lumineux dans un village et leurs sources de sang ont été identifiées par PCR et par le séquençage des gènes du cytochrome *b*.

Résultats: Dans une grotte proche, 65,2 % à 79,8 % des phlébotomes collectés étaient des mâles. Dans un élevage de lapins, 92,9 % à 98,8 % étaient des femelles, tout comme 61,1 % des échantillons collectés dans des porcheries. Certaines femelles portaient des résidus de sang visibles. Le taux de nourrissage était de 49,4 % pour les femelles des porcheries, 12,3 % dans la grotte et seulement 1,7 % dans l'élevage de lapins. Du sang de porc, de lapin, de poulet, de chien et d'humain a été identifié dans les spécimens nourris. Le sang de porc, présent dans tous les échantillons testés, était privilégié, tandis que du sang de chien et de poulet était présent dans un tiers des échantillons.

Conclusion: Dans la circonscription de Jiuzhaigou, dans la province chinoise du Sichuan, la densité considérable de phlébotomes et leur comportement alimentaire péridomestique augmentent le risque de transmission de la leishmaniose viscérale. On pourrait avoir recours à des pulvérisations d'insecticide dans les étables et abris des animaux afin de réduire la population de phlébotomes autour des sites d’occupation humaine.

Translated from English version into French by Suzanne Assenat, through

TRANSLATORS WITHOUT BORDERS

Экологические ниши и источники крови москитов в эндемических очагах висцерального лейшманиоза в Цзючжайгоу, Сычуань, Китай

Хуэин Чен, Кайли Ли, Хау Ши, Йонг Джанг, Ю Ха, Ян Ванг, Дзиндзин Дзян, Сянгъю Ли, Юбинг Ванг, Дженджоу Ян, Дзяннонг Сю, Ядзун Ма

Краткое содержание

Исходные данные Москит *Phlebotomus chinensis* – главный переносчик инфекции висцерального лейшманиоза (ВЛ) в Китае, с широким географическим распределением. Цзючжайгоу, Сычуань – горный эндемичный район ВЛ в Китае. Долгосрочный эффективный контроль позволил добиться сокращения распространения ВЛ в регионе. С целью оценки текущего состояния москитов и их экологического влияния в регионе летом 2014 и 2015 года было проведено исследование.

Методы Образцы москитов собирались при помощи световых ловушек в деревне, источники крови определялись при помощи ПЦР и секвенирования *b* генов митохондриального цитохрома *b*.

Результаты 65.2%-79.8% собранных в ближайшей пещере москитов были женскими особями. 92.9% - 98.8% образцов, собранных на кроличьей ферме, были женскими особями. На свиноводческой ферме 61.1% образцов были женскими особями. У некоторых женских особей были видимые остатки крови. Интенсивность питания составила 49.4% на свиноводческих фермах, 12.3% в пещере и всего 1.7% на кроличьей ферме. В образцах питания была обнаружена свиная, куриная, собачья и человевеческая кровь. Предпочтительным источником крови была свиная кровь, присутствовавшая во всех исследовавшихся образцах, между тем куриная и собачья кровь присутствовала в третьей части образцов.

Заключение Значительная плотность москитов и пищевое поведение в местах проживания человека в Цзючжайгоу, китайской провинции Сычуань повышают риск распространения ВЛ, и распыление инсектицидов в сараях для животных может быть использовано для сокращения популяций москитов в местах проживания людей.

Translated from English version into Russian by Jekaterina Merkuljeva, through

TRANSLATORS WITHOUT BORDERS

Nichos ecológicos y fuentes de sangre del tábano en foco endémico de leishmaniasis visceral en Jiuzhaigou, Sichuan, China

Huiying Chen, Kaili Li, Hua Shi, Yong Zhang, Yu Ha, Yan Wang, Jinjin Jiang, Xiangyu Li, Yubin Wang, Zhenzhou Yang, Jiannong Xu, Yajun Ma

Resumen

Antecedentes El tábano *Phlebotomus chinensis* es el principal vector para la leishmaniasis visceral (LV) en China, con amplia distribución geográfica. Jiuzhaigou, Sichuan es una zona montañosa endémica de LV en China. Los efectivos esfuerzos de control a largo plazo han disminuido con éxito la transmisión de la LV. En el verano de 2014-2015 se llevó a cabo una encuesta para evaluar el estado actual de los tábanos y sus aspectos ecológicos.

Métodos Se recolectaron especímenes de tábanos con trampas de luz en una aldea y se identificaron las fuentes de sangre mediante CRP y secuenciación de genes de citocromo *b* mitocondrial.

Resultados En una cueva cercana, 65,2% a79,8% de los tábanos recolectados fueron machos. En una granja de conejos, 92,9% a98,8% de los especímenes fueron hembras. En las jaulas de cerdos, 61,1% de los especímenes fueron hembras. Algunas hembras tenían residuos de sangre visibles. La tasa de alimentación fue de 49,4% en las hembras en las jaulas de cerdos, 12,3% en las de la cueva, y solo 1,7% en las de la granja de conejos. Se detectó

sangre de cerdo, conejo, pollo, perro y humana en los especímenes alimentados. La sangre de cerdo, presente en todas las muestras evaluadas, fue una fuente de sangre predilecta, mientras que la sangre de pollo y de perro estuvo presente en un tercio de las muestras.

Conclusión En el condado de Jiuzhaigou, en la provincia China de Sichuan, la significativa densidad de tábanos y el comportamiento de alimentación peridoméstica aumentan el riesgo de transmisión de la LV y el rociado con insecticidas en los cobertizos podría utilizarse para reducir la población de tábanos en los entornos humanos.

Translated from English version into Spanish by Maria Alejandra Aguada, through

TRANSLATORS
WITHOUT BORDERS
